# Observational study of characteristics and clinical outcomes of Dutch patients with tuberous sclerosis complex and renal angiomyolipoma treated with everolimus

**DOI:** 10.1371/journal.pone.0204646

**Published:** 2018-11-15

**Authors:** Bernard A. Zonnenberg, Maureen P. Neary, Mei Sheng Duh, Raluca Ionescu-Ittu, Jonathan Fortier, Francis Vekeman

**Affiliations:** 1 University Medical Center Utrecht, Utrecht, Netherlands; 2 Novartis Pharmaceuticals Corporation, East Hanover, NJ, United States of America; 3 Analysis Group, Inc., Boston, MA, United States of America; 4 Groupe d’analyse, Ltée, Montréal, QC, Canada; University of Catanzaro, ITALY

## Abstract

**Objective:**

To compare kidney size (used as proxy for total renal angiomyolipoma [rAML] size) and kidney function outcomes between patients with tuberous sclerosis complex (TSC) and rAML treated and not treated with everolimus.

**Methods:**

Medical charts of adults with TSC-associated rAML followed at a specialty medical center in the Netherlands (1990–2015). Included patients treated with everolimus (n = 33, of which 27 were included in the kidney size analyses and 27 in the kidney function analyses [21 patients in both]; index date = everolimus initiation) and non-treated patients (n = 39, of which 29 were included in the kidney size analyses and 33 in the kidney function analyses [23 patients in both]; index date = one date among all dates with outcome measurement).Percent change in kidney size and kidney function from the index date to the best measurement in the two years post-index date (best response) compared between patients treated and not treated with everolimus.

**Results:**

Compared with non-treated patients, significantly more everolimus-treated patients experienced a reduction in the size of their largest kidney in the two years post-index date (85.2% vs. 37.9%, p < 0.01). Also, there was a tendency towards more improvement in the estimated glomerular filtration rate (eGFR) among the everolimus-treated patients (55.6% vs. 33.3%, p = 0.08).

**Conclusions:**

The study results suggest that everolimus is effective in controlling and even reversing the growth of the kidneys, used as a proxy for rAML size, as well as preserving or improving kidney function in patients with TSC and rAML treated in a real-world, observational setting.

## Introduction

Tuberous sclerosis complex (TSC) is a disorder characterized by the growth of non-malignant tumors in various body organs [1]. Approximately 70–85% of patients with TSC form renal angiomyolipomas (rAML) [2–5]. Large rAMLs may be associated with acute flank pain, potentially life-threatening hemorrhage due to ruptured aneurysms, and impaired renal function [6]. Thus, preserving renal function and minimizing the risk of complication are the main therapeutic goals for patients with TSC and rAML (TSC-rAML) [7]. Arterial embolization, resection, and partial/total nephrectomy are typically reserved for large rAMLs and for cases of severe hemorrhage or vascular invasion [8–10], while mammalian target of rapamycin inhibitors (mTORi) can be used to reduce tumor size and prevent rAML growth in remaining patients [11–13]. Everolimus, an mTORi approved both in the United States (US) and Europe for the treatment of TSC-associated rAML [14, 15], was shown in the phase III, EXIST-2 trial to reduce total rAML volume significantly more than placebo while exhibiting an acceptable safety profile [16]. However, real-world studies are needed to confirm these results. The current study used medical chart data from a specialty clinic in the Netherlands to compare kidney size, used as proxy for total rAML size, and kidney function between patients with TSC-associated rAML treated and not treated with everolimus.

## Materials and methods

### Data source

Medical charts of patients with TSC treated from 01/01/1990 to 31/12/2015 at University Medical Center Utrecht (UMCU), a kidney tumor major specialty center in the Netherlands, were reviewed.

TSC diagnosis was based on the Revised 1998 Criteria (i.e., presence of 2 major criteria or 1 major and 2 minor criteria [17]). All patients provided written consent to participate in the study. Data extracted from the patients' medical records for the analysis were de-identified, but not fully annonymized. The study was approved by the UMCU institutional review board (study METC 14/412C).

### Patients

**Fig 1:**
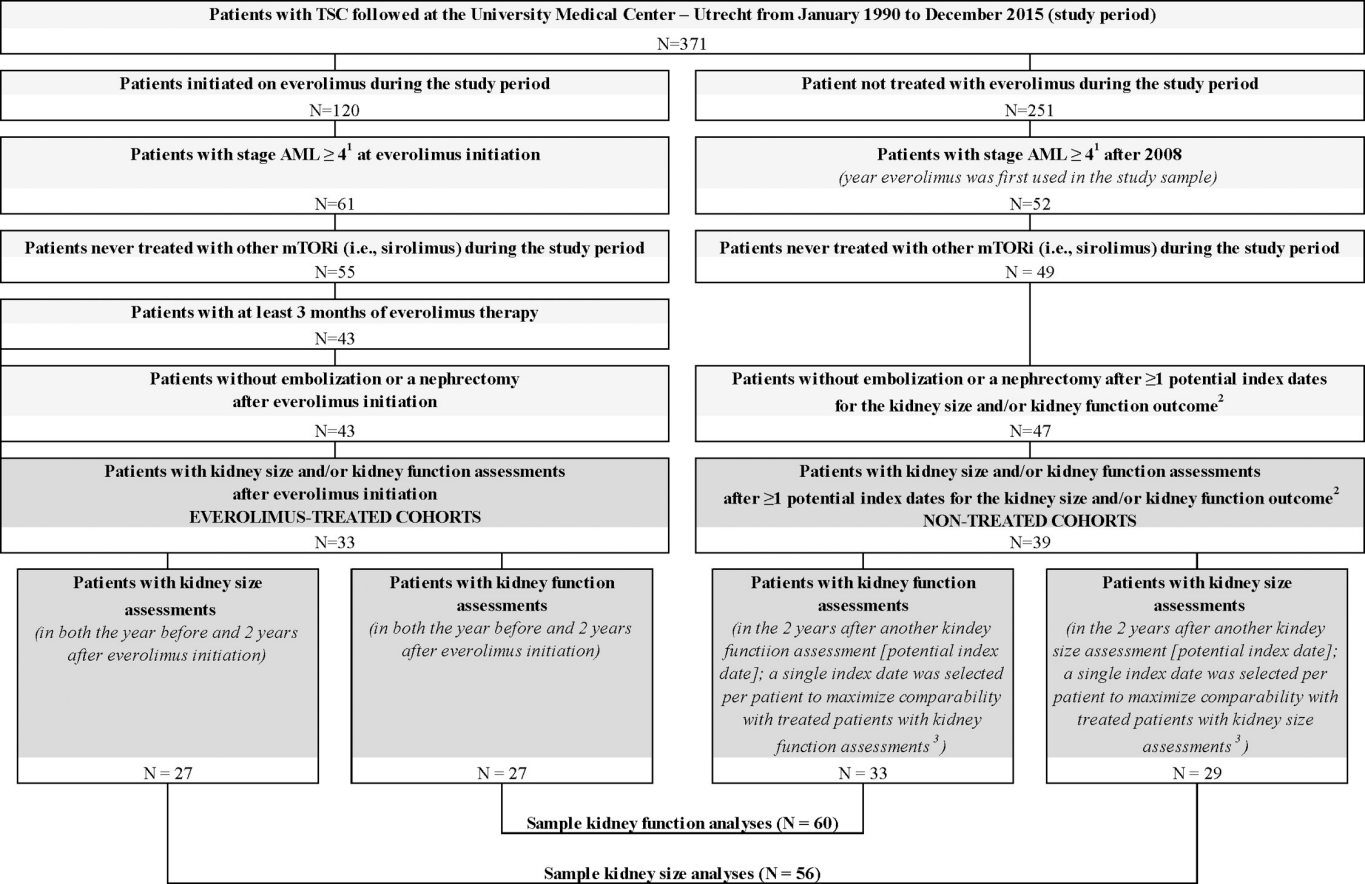
Patient disposition. ^1^ Please see Table 1 for criteria used to define rAML stage. ^2^ For the kidney size outcome, potential index dates were selected from days with kidney size assessments (with an MRI or CT sca); for the kidney function outcome potential index dates were selected from days with creatinine assessments. ^3^ For a given patient and a given outcome, if multiple potential index dates were available, a single index date was selected based on time at which the patient was the most likely to be treated with everolimus (probability of everolimus treatment was estimated from a logistic regression model including the following covariates: age at index date and eGFR and size of the largest kidney at the index date).

Of 371 patients with TSC-associated rAML managed at UMCU, 33 everolimus-treated patients and 39 patients not treated with mTORi were included in the study (Fig 1). This study is non-interventional and utilizes pre-existing data derived from the course of patient care at UMCU. The decision to treat patients with mTORi (i.e., sirolimus and/or everolimus), or to use embolization or surgery during patient care was determined by patients’ treating physicians, including the first author of the current study. While individual physician practices may vary, patients who were prescribed mTORi at UMCU before the mTORis' approval by the European Medicines Agency for TSC indication were generally those not eligible for more standard options, e.g., when previous embolization was found to be not effective or when the vasculature of the patient made embolization impossible.

All patients had kidney size and/or kidney function assessments both in the year before and two years after an index date defined as (a) date of everolimus treatment initiation for everolimus-treated patients, and (b) date with kidney size or kidney function assessment (depending on the analysis) for non-treated patients. Patients with embolization or nephrectomy after the index date were excluded from the current study as these procedures could have confounded the kidney size and kidney function measurements post-index. When multiple potential index dates were available for a given outcome, logistic regression-based propensity scores models to select a single index date corresponding to the date when the patient had the highest propensity of receiving everolimus treatment. Since everolimus was first used for TSC at UMCU in 2008, the index date had to be in 2008 or afterwards. Because everolimus use in patients with rAML stage < 4 may target non-renal TSC manifestations, patients were also required to have rAML stage ≥ 4 at the index date (Table 1). Of 33 everolimus-treated patients satisfying the inclusion criteria, 27 were included in the kidney size analyses and 27 in the kidney function analyses (21 patients included in both); of 39 non-treated with mTORi patients satisfying the inclusion criteria, 29 and 33 were included in the kidney size and kidney function analyses, respectively (23 patients included in both; Fig 1). Patients included in the analyses had no nephrectomy or embolization at index or post-index. Outcomes were measured from index up to two years post-index, upon data availability.

**Table 1:** Renal angiomyolipoma staging criteria.

Stage^1^	No. of rAMLs	rAML size	Kidney anatomy
0		None^2^	Normal
1	≤ 5	1 cm to 3.5 cm	Normal
2	˃ 5	1 cm to 3.5 cm	Normal
3	≤ 5	At least 1 rAML ≥ 3.5 cm	Kidney intact
4	˃ 5	1 to 4 rAMLs ≥ 3.5 cm	Kidney intact
5	˃ 5	5 or more rAMLs ≥ 3.5 cm	Kidney recognizable
6	˃ 5	At least 1 rAML ≥ 5.0 cm	Kidney not recognizable

rAML: renal angiomyolipoma.

^1^ All three conditions need to be satisfied.

^2^ Not able to definitely determine via CT scan if lesions < 1 cm in longest diameter are angiomyolipoma.

### Outcomes

Study outcomes were defined as the change in kidney size and kidney function from the index date (or most recent value in the year pre-index) to the best value in the two years post-index (best response, similar to the EXIST-2 trial [17]). Outcomes were reported as percentage change relative to the baseline value to account for potential differences between patients at baseline. Kidney size was defined as the coronal size of the largest kidney, to account for patients who may have had a kidney nephrectomy. Kidney size was evaluated using enhanced computed tomography/magnetic resonance imaging as part of routine care. The kidney size outcome was used as proxy for the total rAML size (i.e., outcome of the EXIST-2 trial [16] which could not be evaluated from available information in patients’ medical charts due to the advanced stage of the disease). Kidney function was defined based on glomerular filtration rate (eGFR) estimated from routine creatinine lab results using the chronic kidney disease epidemiology collaboration (CKD-EPI) formula [18]. The best response in eGFR was stratified by CKD stage to account patients who may have had a decrease in eGFR while remaining in the normal limits of their kidney function. For everolimus-treated patients, both outcomes were also stratified by duration of treatment with everolimus (i.e., <12 versus ≥12 months). Time from index to best response was also described for both outcomes.

### Statistical analysis

Analyses were performed separately for each outcome. Patient characteristics and outcomes were described using descriptive statistics. Where applicable, outcomes were compared between cohorts using Fisher, chi-square, or t-tests. Statistical analyses were performed using SAS statistical software, version 9.3 (SAS Institute, Cary, NC).

## Results

### Kidney size

Among the 27 everolimus-treated patients with kidney size assessments, treatment duration ranged from 3.6 to 36.0 months, with 81.5% treated for ≥ 12 months (Table 2). Patient characteristics were largely similar between the treated and non-treated cohorts at the index date: mean age 42.1 and 42.9 years, females 48.1% and 55.2%, hypertension 7.4% and 6.9%, mean eGFR pre-index date 100.4 and 101.1 mL/min/1.73m^2^, and stage 1 CKD 63.0% and 72.4%, respectively (Table 3). In both cohorts, all patients were White. While some differences were observed between the treated and non-treated cohorts in SEGA (44.4% vs. 17.2%), LAM (11.1% vs. 20.7%), and embolization (55.6% vs. 69.0%; Table 3), the baseline kidney size was similar in the treated and non-treated cohorts (mean coronal size of the largest kidney 139.7 mm vs. 135.9 mm, p = 0.6271; Table 4) and there were no statistically significant differences between cohorts in the rAML stage (p = 0.2101; Table 4).

**Table 2:** Characteristics of everolimus therapy.

Characteristics of everolimus therapy	Everolimus-treated patients			
	With kidney size assessments^1^		With kidney function assessments^2^	
	(N = 27)		(N = 27)	
**Year everolimus was initiated**				
<2013	1	(4)	1	(4)
2013	7	(26)	7	(26)
2014	16	(59)	13	(48)
2015	3	(11)	6	(22)
**Time to initiation of everolimus (years), mean ± SD [median]**				
From first rAML assessment	9.0 ± 3.0	[10.0]	9.2 ± 3.2	[10.3]
From first rAML assessment of stage ≥ 4	7.5 ± 3.5	[7.9]	7.6 ± 3.6	[8.0]
**Duration of therapy with everolimus**^3^ **(months)**				
Mean ± SD [median]	18.4 ± 9.8	[14.5]	17.6 ± 10.8	[13.8]
Min—max range	3.6–36.0		5.7–36.0	
Duration categories, n (%)				
< 3 months	-		-	
3 to 6 months	2	(7)	2	(7)
6 to 12 months	3	(11)	7	(26)
> 12 months	22	(82)	18	(67)
**Patients still on the everolimus at data cutoff, n (%)**	24	(89)	24	(89)

SD: standard deviation; rAML: renal angiomyolipoma.

^1^ To be eligible, a patient was required to have ≥ 1 evaluation of the kidney size (with an MRI or a CT scan) in the one year period prior to index date and ≥ 1 evaluation in the two year period after index date.

^2^ To be eligible, a patient was required to have ≥ 1 creatinine measurement in the one year period prior to index date and ≥ 1 creatinine measurement in the two year period after index date.

^3^ From the first everolimus dispensing to first occurring between end of therapy with everolimus, death, or last day of observation.

**Table 3:** Patient demographics and clinical characteristics at index date or pre-index.

	Patients with kidney size assessments^1^				Patients with kidney function assessments^2^			
Characteristics at index date	Patients initiated on everolimus		Patients not treated with an mTORi		Patients initiated on everolimus		Patients not treated with an mTORi	
	(N = 27)		(N = 29)		(N = 27)		(N = 33)	
**Patient age (years)**								
Mean ± SD [median]	42.1 ± 11.6	[41.1]	42.9 ± 12.7	[43.6]	39.6 ± 9.5	[40.0]	44.0 ± 11.0	[43.9]
Age categories, n (%)								
< 18 years	-		-		-		-	
18 to 24 years	1	(4)	2	(7)	1	(4)	1	(3)
25 to 34 years	6	(22)	6	(21)	6	(22)	6	(18)
35 to 44 years	12	(44)	10	(35)	14	(52)	13	(39)
45 to 54 years	6	(22)	7	(24)	5	(19)	7	(21)
55 to 64 years	-		3	(10)	-		6	(18)
≥ 65 years	2	(7)	1	(3)	1	(4)	-	
**Female, n (%)**	13	(48)	16	(55)	13	(48)	19	(58)
**Gene mutation, n (%)**								
Yes	18	(67)	12	(41)	18	(67)	13	(39)
* TSC1*	2	(7)	2	(7)	2	(7)	1	(3)
Deletion	1	(4)	1	(3)	1	(4)	-	
Transition	1	(4)	1	(3)	1	(4)	1	(3)
* TSC2*	16	(5)	10	(35)	16	(59)	12	(36)
Insertion	-		1	(3)	-		1	(3)
Deletion	6	(22)	5	(17)	5	(19)	3	(9)
Duplication	2	(7)	-		3	(11)	-	
Transversion	4	(15)	1	(3)	3	(11)	2	(6)
Transition	4	(15)	3	(10)	5	(19)	6	(18)
No identified mutation	2	(7)	6	(21)	4	(15)	6	(18)
Unknown	7	(26)	11	(38)	5	(19)	14	(42)
**Hypertension**^3^**, n (%)**								
* *Yes	2	(7)	2	(7)	3	(11)	5	(15)
* *No	6	(22)	6	(21)	8	(30)	13	(39)
* *Unknown	19	(70)	21	(72)	16	(59)	15	(46)
**SEGA, n (%)**								
* *Yes	12	(44)	5	(17)	13	(48)	8	(24)
Definitely	4	(15)	1	(3)	4	(15)	2	(6)
Likely	2	(7)	1	(3)	2	(7)	3	(9)
Possible	6	(22)	3	(10)	7	(26)	3	(9)
* *Unlikely	-		-		-		-	
* *Unknown	15	(56)	24	(83)	14	(52)	25	(76)
**LAM, n (%)**								
* *Yes	3	(11)	6	(21)	3	(11)	9	(27)
Mild	2	(7)	2	(7)	3	(11)	4	(12)
Moderate	1	(4)	3	(10)	-		3	(9)
Severe	-		1	(3)	-		2	(6)
* *No	11	(41)	10	(35)	14	(52)	12	(36)
* *Unknown	13	(48)	13	(45)	10	(37)	12	(36)
**Prior surgery, n (%)**								
* *Embolization	15	(56)	20	(69)	16	(59)	25	(76)
* *Nephrectomy	-		1	(3)	1	(4)	1	(3)
* *Kidney transplant	-		-		-		-	
**Pre-index coronal size of largest kidney, mean ± SD [median]**	139.7 ± 33.8	[131.0]	135.9 ± 24.1	[136.0]	145.5 ± 42.3	[131.0]	140.5 ± 36.9	[130.0]
**Pre-index eGFR (mL/min/1.73m**^**2**^**), mean ± SD [median]**	100.4 ± 27.4	[108.0]	101.1 ± 26.1	[106.6]	101.9 ± 30.7	[111.0]	99.4 ± 26.2	[104.5]
**CKD stage, n (%)**								
* *eGFR ≥ 90 (normal eGFR)	17	(63)	21	(72)	18	(67)	27	(82)
* *eGFR 60 to 89 (stage 2)	7	(26)	6	(21)	5	(19)	3	(9)
* *eGFR 45 to 59 (stage 3A)	3	(11)	2	(7)	4	(15)	3	(9)

CKD: chronic kidney disease; mTORi: mammalian target of rapamycin inhibitor; SD: standard deviation; LAM: lymphangioleiomyomatosis; SEGA: subependymal giant cell astrocytoma.

^1^ To be eligible, a patient was required to have ≥ 1 evaluation of the kidney size (with an MRI or a CT scan) in the one year period prior to index date and ≥ 1 evaluation in the two year period after index date.

^2^ To be eligible, a patient was required to have ≥ 1 creatinine measurement in the one year period prior to index date and ≥ 1 creatinine measurement in the two year period after index date.

^3^ Defined as systolic pressure ≥ 140 mmHg or diastolic pressure ≥ 90 mmHg within 180 days before index date. For patients with multiple blood pressure measurements within this period, hypertension status is based on the measurement closest to everolimus initiation date.

**Table 4:** Kidney size measurements pre- and post-index date.

	Patients initiated on everolimus^1^		Patients not treated with an mTORi^1^		P-value^2^
	(N = 27)		(N = 29)		
**Pre-index date**	** **	** **	** **	** **	** **
Coronal size of the largest kidney^3^ (mm), mean ± SD [median]	139.7 ± 33.8	[131.0]	135.9 ± 24.1	[136.0]	0.63
rAML stage^3^, n (%)					
* *4	20	(74)	19	(66)	0.21
* *5	2	(7)	7	(24)	
* *6	5	(19)	3	(10)	
**Post-index date**	** **	** **	** **	** **	
Best response in coronal size of the largest kidney during the first 2 years following the index date^4^					
* *Kidney reduction^5^, n (%)	23	(85)	11	(38)	< 0.001
* *Kidney increase^5^, n (%)	4	(15)	12	(41)	
* *No changes in kidney size^5^, n (%)	-		6	(20)	
Magnitude of kidney size change (mm) for the best response^4^ in coronal size of the largest kidney during the first 2 years following the index date, mean ± SD [median]	−8.8 ± 8.9	[−8.0]	−1.7 ± 11.6	[0.0]	0.01
* *Among patients with kidney reduction^5^	−11.2 ± 7.1	[−13.0]	−12.6 ± 9.7	[−11.0]	0.63
* *Among patients with kidney increase^5^	5.0 ± 3.7	[4.5]	7.6 ± 6.4	[5.5]	0.46
Time from index date to best response^4^ (months), mean ± SD [median]	8.2 ± 5.0	[8.7]	14.1 ± 6.1	[14.9]	0.0003

SD: standard deviation; mTORi: mammalian target of rapamycin inhibitor; rAML: renal angiomyolipoma.

^1^ To be eligible, a patient was required to have ≥ 1 evaluation of the kidney size (with an MRI or a CT scan) in the one year period prior to treatment initiation and ≥ 1 evaluation in the two year period after index date.

^2^ Calculated using Pearson chi-square or Fisher tests for categorical variables and two sided t-tests for continuous variables.

^3^ Most recent value in the one year period prior to index date.

^4^ Kidney size assessment with the lowest value during the 2-years after index date.

^5^ Change in kidney size from the most recent value in the one year period prior to index date to the lowest value during the 2-years after index date.

**Fig 2:**
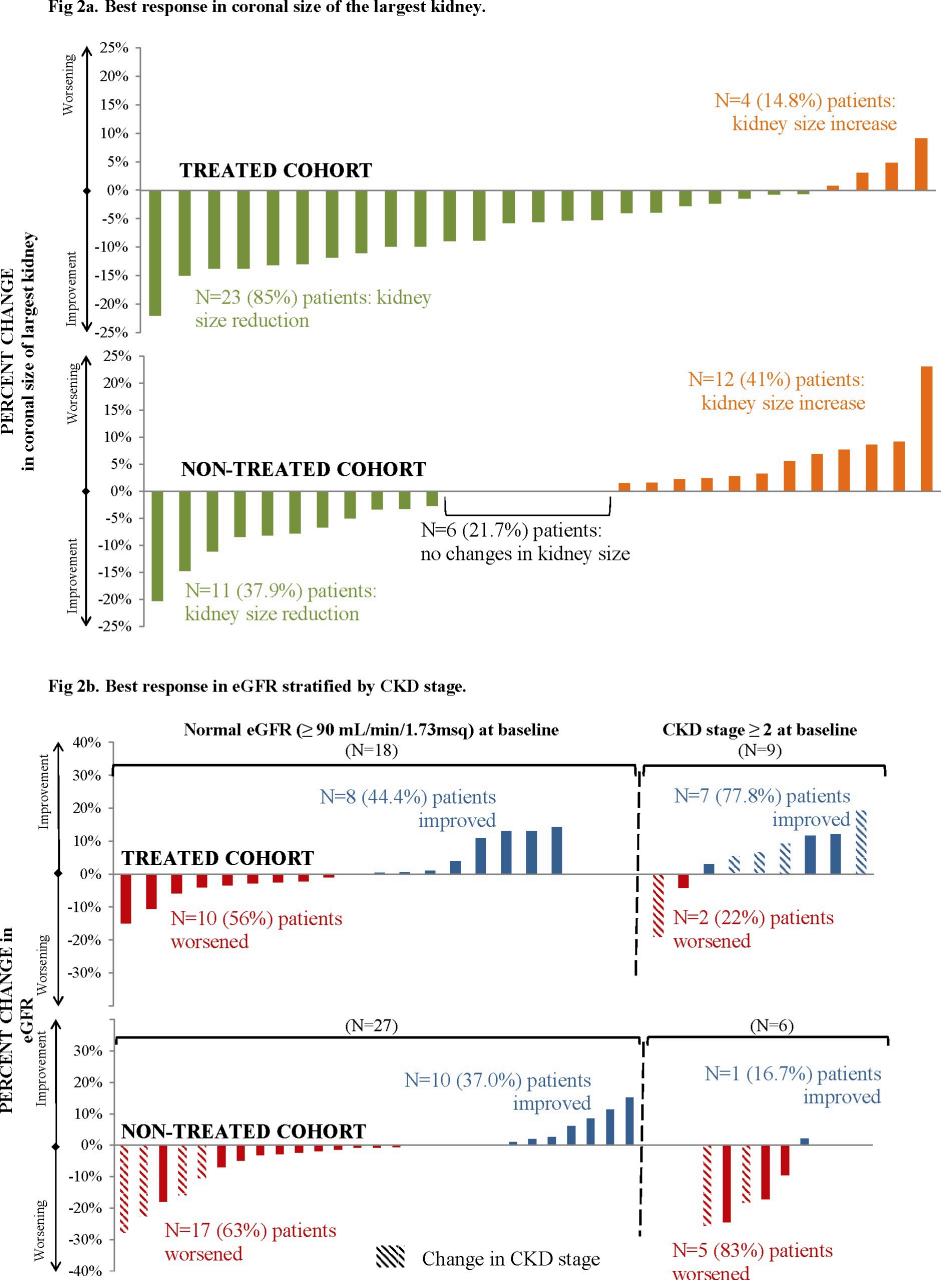
Best response in outcomes in treated versus non treated patients. **a. Best response in coronal size of the largest kidney. b. Best response in eGFR stratified by CKD stage.** eGFR: estimated glomerular filtration rate; CKD: chronic kidney disease.

In the two years post-index (median observation period: 13 months for everolimus-treated patients vs. 24 months for non-treated patients), significantly more everolimus-treated patients experienced a kidney size reduction compared with non-treated patients (85.2% vs. 37.9%; p = 0.0003). The average change in kidney size was -8.8 mm for everolimus-treated patients and -1.7 mm for non-treated patients (p = 0.01). Among patients with a kidney size reduction, the magnitude of the reduction was similar in the two cohorts (mean 11.2 vs. 12.6 mm, p = 0.6327; Table 4 and Fig 2A). The mean time to best response in kidney size was significantly shorter for the everolimus-treated patients compared with non-treated patients (8.2 vs. 14.1 months, p = 0.0003; Table 4). Among everolimus-treated patients, no difference in the proportion of patients with a kidney size reduction was found between patients treated <12 versus ≥12 months (80.0% vs. 86.4%, p = 0.72).

### Kidney function

Among the 27 everolimus-treated patients with kidney function assessments, treatment duration ranged from 5.7 to 36.0 months, with 66.7% treated for ≥ 12 months (Table 2). Patient characteristics were largely similar between the treated and non-treated cohorts at the index date: mean age 39.6 and 44.0 years (Table 3), stage 4 rAMLs 66.7% and 75.9%, and the mean coronal size of the largest kidney 145.5 mm and 140.5 mm, respectively. In both cohorts, all patients were White. While some differences were observed between the treated and non-treated cohorts in gender (females 48.1% vs. 57.6%), SEGA (48.1% vs. 24.2%), LAM (11.1% vs. 27.3%), and embolization (59.3% vs. 75.8%; Table 3), the baseline eGFR was similar in the treated and non-treated cohorts (mean eGFR 101.9 and 99.4 mL/min/1.73m^2^, p = 0.7299) and there were no statistically significant differences between cohorts in the proportion of patients with CKD of different stages (p = 0.3867; Table 5).

**Table 5:** Kidney function measurements pre- and post-index date.

	Patients initiated on everolimus^1^		Patients not treated with an mTORi^1^		P-value^2^
	(N = 27)		(N = 33)		
**Pre-index date**					
eGFR^3^^,^^4^ (mL/min/1.73m^2^), mean ± SD [median]	101.9 ± 30.7	[111.1]	99.4 ± 26.2	[104.5]	0.73
CKD stage,^3^^,^^4^ n (%)					
* *eGFR ≥ 90 (normal eGFR)	18	(67)	27	(82)	0.39
* *eGFR 60 to 89 (stage 2)	5	(19)	3	(9)	
* *eGFR 45 to 59 (stage 3A)	4	(15)	3	(9)	
**Post-index date**					
Best response in eGFR during the first 2 years following index date^5^					
* *eGFR improvement^6^, n (%)	15	(56)	11	(33)	0.08
* *eGFR worsening^6^, n (%)	12	(44)	22	(67)	
Magnitude of the eGFR change (mL/min/1.73m^2^) for the best response^5^ in eGFR during the first 2 years following index date, mean ± SD [median]	0.9 ± 8.9	[0.7]	−3.9 ± 9.5	[−1.7]	0.05
* *Among patients with eGFR improvement^6^	6.9 ± 5.1	[5.8]	4.5 ± 5.1	[2.1]	0.25
* *Among patients with eGFR worsening^6^	−6.6 ± 6.6	[−4.1]	−8.1 ± 8.3	[−5.1]	0.60
Stratification by CKD stage at index date for the best response^5^ in CKD stage during the first 2 years following index date					
* *Among patients with normal eGFR at index date	n = 18		n = 27		
* *CKD stage worsened^6^, n (%)	-		4	(15)	<0.001
* *CKD stage remained the same^6^, n (%)	18	(100)	23	(85)	
* *Among patients with CKD stage ≥ 2 at index date	n = 9		n = 6		
CKD stage improved^6^, n (%)	4	(44)	-		0.16
CKD stage worsened^6^, n (%)	1	(11)	2	(33)	
CKD stage remained the same^6^, n (%)	4	(44)	4	(67)	
Time from index date to best response in eGFR (months), mean ± SD [median]	8.5 ± 6.5	[7.0]	10.2 ± 7.0	[11.0]	0.34

SD: standard deviation; eGFR: estimated glomerular filtration rate; CKD: chronic kidney disease; mTORi: mammalian target of rapamycin inhibitor.

^1^ To be eligible, a patient was required to have ≥ 1 creatinine measurement in the one year period prior to index date and ≥ 1 creatinine measurement in the two year period after index date.

^2^ Calculated using Pearson chi-square or Fisher tests for categorical variables and two sided t-tests for continuous variables.

^3^ Most recent value in the one year period prior to index date.

^4^ Estimated using the CKD-EPI equation.

^5^ eGFR assessment with the highest value during the 2-years after index date.

^6^ Change in eGFR from the most recent value in the one year period prior to index date to the lowest value during the 2-years after index date.

In the two years post-index, there was a more pronounced tendency towards less eGFR worsening among the everolimus-treated patients (median observation period: 12 months) compared with the non-treated patients (median observation period: 24 months) but the difference did not reach statistical significance (44.4% vs. 66.7%, p = 0.0840; Table 5). Among 45 patients with normal kidney function at baseline, significantly fewer everolimus-treated patients had a worsening of the CKD stage compared with non-treated patients (0% vs. 14.8% with worsening, p < 0.0001); among 15 patients with CKD stage ≥ 2 at baseline, everolimus-treated patients appeared to have greater improvement in CKD stage than non-treated patients, but the difference was not statistically significant (44.4% vs. 0% with improvement, p = 0.1552; Table 5 and Fig 2B). The mean time to best response in kidney function was not significantly different between the cohorts (p = 0.3417; Table 5). Among everolimus-treated patients, no difference in the proportion of patients with a worsening eGFR was found between patients treated <12 versus ≥12 months (55.6%% vs. 38.9%, p = 0.41).

## Discussion

Using a real-world sample of patients with TSC-associated rAML treated at a specialty center in the Netherlands, the study showed that significantly more everolimus-treated patients experienced a size reduction of their largest kidney in the two years after initiating treatment compared with patients who were not treated over a similar time period (85.2% vs. 37.9%, p = 0.0003). In 37% of the everolimus-treated patients, the kidney size reduction amounted to ≥ 10%. More everolimus-treated patients also experienced an improvement in eGFR compared with non-treated patients (55.6% vs. 33.3%). These results strongly suggest that everolimus can lead to a reduction in kidney size–and thus potentially in rAML volume–, the preservation and improvement of kidney function, and the protection of organ loss.

Real-world findings from the current study substantiate the results of the phase III EXIST-2 trial, in which rAML volume was reduced in 42% of everolimus-treated patients versus 0% of placebo-controlled patients [16]. The percentage of patients experiencing rAML volume or kidney size reduction may be different in the current study, compared with the EXIST-2 trial, due to design differences (observational study vs. clinical trial), sample differences (patients with ≥ 5 rAMLs with ≥ 1 rAML ≥ 3.5 cm in diameter in the current study vs. ≥ 1 rAML ≥ 3 cm in diameter in EXIST-2), and differences in the outcome measurement (kidney size reduction vs. a ≥ 50% reduction in the sum of volumes of target rAMLs). To our knowledge, only one study in a real-world setting investigated the impact of everolimus treatment on rAML volume. Using a sample of Asian patients, Hatano et al. found that 68% (32/47) everolimus-treated patients with TSC-associated rAMLs experienced a ≥ 50% reduction in rAML volume within 6 months of treatment initiation and 98% (46/47) experienced an rAML volume reduction (mean = 60% reduction) over a median treatment duration of 14 months [19]. The effect of everolimus appeared to be greater in Hatano et al. than in the current study, possibly due to differences in the population investigated (Asian vs. White), sample selection (patients with rAML of ≥ 4 cm and no severe forms of epilepsy or LAM vs. all TSC-rAML patients) and outcome measurement (rAML volume vs. coronal size of largest kidney). Notwithstanding these differences, evidence from both studies suggest a link between everolimus treatment and kidney/rAML reduction in TSC patients. Moreover, the present study suggested that everolimus may be beneficial even to TSC patients with more advanced rAML, extending results from the EXIST-2 trial [16].

In the current study, 37.9% of non-treated patients experienced kidney size reduction. Although unexpected, this could be explained by several factors. First, measurement variations may occur between consecutive assessments in the same patient [20]. Indeed, most non-treated patients experienced very modest kidney size reductions (73% had ≤ 10% reduction), supporting this hypothesis. Importantly, such variations are likely to affect treated and non-treated cohorts to the same extent and are not expected to impact cohort comparisons. Second, renal insufficiency due to kidney vasculitis or glomerulonephritis may also lead to kidney size reductions. While these diagnoses were not available in the data, they usually represent rare occurrences and are unlikely to affect study results. It is also important to note that among non-treated patients having a kidney size reduction, the time to response was significantly longer by 5.9 months compared with the time to response of treated patients. Similarly, the study finding that 33.3% of non-treated patients experienced an improvement in eGFR could be explained by natural variations in creatinine in response to meals, exercise, posture, changes in blood pressure, and other conditions [21]. As observed for kidney size reduction, among non-treated patients having an eGFR improvement, the time to response was also longer by 1.7 months compared with the time to response of treated patients.

A distinctive feature of the present study is the assessment of kidney function following everolimus treatment, an outcome not included in the EXIST-2 trial. Findings of this study showed a tendency towards less worsening, and possibly improvement, in kidney function in everolimus-treated patients compared with non-treated patients, even though differences between the two cohorts did not reach statistical significance. However, it is very possible that the current study lacked the statistical power to detect small, but clinically meaningful differences. The results of the EXIST-2 trial [16] current study, and Hatano et al. [19] support the hypothesis that impact of everolimus on kidney function is mediated through its impact on rAML volume and kidney size. However, everolimus may also have a direct effect on kidney function. Indeed, previous studies have shown that everolimus inhibits smooth muscle cell proliferation and prevents vascular remodeling [22], which is one of the manifestations of CKD [23]. Furthermore, some of the results from the current study also support a direct effect of everolimus on kidney function: none of the everolimus-treated patients with CKD starting treatment in CKD stage 1 experienced a decline in kidney function (versus 14.8% of non-treated patients with CKD stage 1) and almost 45% of those starting treatment in CKD stage ≥ 2 saw their kidney function improve (versus 0% of non-treated patients with CKD). Further studies including both rAML volume and kidney function as study endpoints are warranted to verify these hypotheses.

Overall, the current study provides real-world evidence of the relationship between everolimus and kidney size reduction in patients with TSC-associated rAML in a more advanced stage than previously reported. Furthermore, it also provides evidence of a potential link between everolimus and improved kidney function. By reducing rAML volume [16] and kidney size and by preserving or improving kidney function, everolimus and other mTORi may significantly advance the treatment of TSC-associated rAML. Indeed, since the risk of complications (particularly bleeding) increases with the size of the rAMLs and rAMLs can regrow after embolization [24, 25] everolimus may also lead to a reduced risk of surgery and hospitalization and an improved quality of life for these patients. Further studies with larger sample size and longer follow-up are needed to corroborate and extend results of the present study.

Several limitations should be considered. First, this study included patients followed at a specialty center and results might not be generalizable to the entire TSC population. Second, kidney size was used as a proxy for total rAML size and the extent to which the two measures correlate is unknown. However, rAMLs located on the upper/lower kidney poles are likely captured well by the kidney size measure. While presence of renal cysts may also influence the kidney size, current evidence shows that everolimus has no effect on cysts in patients with TSC [26]. Therefore, the differences between everolimus-treated and untreated in kidney size changes from baseline observed in the current study is likely related to a change in the size of renal angiomyolipomas and not a change in cysts size. Third, because everolimus was approved recently, an important confounder may be that everolimus-treated patients had shorter median follow-up than untreated patients (13 vs. 24 months for kidney size and 12 vs. 24 months for kidney function). However, this unbalance was offset by the fact that treated patients underwent more frequent kidney size assessments (33% vs. 3% with ≥ 2 assessments post-index in treated vs. non-treated) and approximately the same number of kidney function assessments (52%. vs. 49% with ≥ 2 assessments post-index in treated vs. non-treated). While some patients might have undergone additional laboratory/imaging testing at other centers, the cohorts were likely equally affected. Fourth, the database used in the current study did not include information on bleeding complications, everolimus dosage, or reason for discontinuation of treatment. Lastly, this is a non-randomized study and unmeasured differences between cohorts might exist.

## Conclusions

The results for this longitudinal observational study suggest that everolimus is effective in controlling and even reversing kidney growth, used as a proxy for rAML size, as well as preserving or improving kidney function.
